# Simplified Brain Organoids for Rapid and Robust Modeling of Brain Disease

**DOI:** 10.3389/fcell.2020.594090

**Published:** 2020-10-28

**Authors:** Jeongmin Ha, Ji Su Kang, Minhyung Lee, Areum Baek, Seongjun Kim, Sun-Ku Chung, Mi-Ok Lee, Janghwan Kim

**Affiliations:** ^1^Stem Cell Convergence Research Center, Korea Research Institute of Bioscience and Biotechnology (KRIBB), Daejeon, South Korea; ^2^Department of Functional Genomics, KRIBB School of Bioscience, Korea University of Science and Technology (UST), Daejeon, South Korea; ^3^Mibyeong Research Center, Korea Institute of Oriental Medicine, Daejeon, South Korea

**Keywords:** brain organoid, neural stem cells, disease modeling, drug screening, Parkinson’s disease, LRRK2, gene editing

## Abstract

Although brain organoids are an innovative technique for studying human brain development and disease by replicating the structural and functional properties of the developing human brain, some limitations such as heterogeneity and long-term differentiation (over 2 months) impede their application in disease modeling and drug discovery. In this study, we established simplified brain organoids (simBOs), composed of mature neurons and astroglial cells from expandable hPSC-derived primitive neural stem cells (pNSCs). simBOs can be rapidly generated in 2 weeks and have more homogeneous properties. Transcriptome analysis revealed that three-dimensional (3D) environment of simBOs facilitates the conversion of pNSCs to mature neuronal systems compared to a two-dimensional environment in the context of neurotransmitter release, synaptic vesicle formation, ion channels, calcium signaling, axonal guidance, extracellular matrix organization, and cell cycle. This result was correlated with the translocation of YAP1 into the cytoplasm by sensing matrix stiffness on the 3D models. Furthermore, we demonstrated that simBOs could easily be specified into midbrain-like simBOs by treatment with Shh and FGF8. Midbrain-like simBOs from a Parkinson’s disease patient (*LRRK2*^G2019S^)-derived pNSCs and gene-corrected (*LRRK2*^*WT*^) control pNSCs represented disease-associated phenotypes in terms of increased LRRK2 activity, decreased dopaminergic neurons, and increased autophagy. Treatment with the LRRK2 inhibitor, PFE-360, relieved the phenotype of Parkinson’s disease in midbrain-like simBOs. Taken together, these approaches could be applied to large-scale disease models and alternative drug-testing platforms.

## Introduction

Neurological disorders, an immense threat to human health, are diseases with psychological and/or physical symptoms characterized by an abnormality in the development, patterning, and maintenance of homeostasis of the human brain or nervous system (Thakur et al., 2016). Besides the complexity of the biological structure and function of the human brain, the difficulty of obtaining human brain tissues impedes understanding of pathogenesis and drug discovery. Human neuronal tissues can only be obtained from postmortem or surgically removed brain tissues. In addition, primary neurons derived from the brain are post-mitotic cells, which limits *in vitro* expansion (Koo et al., 2019). The experimental animal models have provided considerable insights into the molecular basis of normal brain development, disease pathogenesis, and therapeutic options for neurological disorders, but the inter-species differences in development (Liu et al., 2012; Homem et al., 2015) and pathogenesis (Burbulla et al., 2017; Gitler et al., 2017) make understanding the human brain and disease challenging. Furthermore, recent advances in single-cell transcriptome revealed divergent gene expression between homolog cell types of human versus mouse cortex associated with neuronal connectivity, signaling, neurotransmitters, and cell-type-specific markers, implying functional variations between the two species (Hodge et al., 2019). Thus, new experimental models are required for replicating the characteristics of the human brain and pathological conditions, in order to better understand the human brain development and disease.

Human pluripotent stem cells (hPSCs) (Thomson et al., 1998; Takahashi et al., 2007) have provided tremendous opportunities to study the human brain. The development of neuronal-cell specific differentiation has made it possible to obtain various types of neural cells (Tao and Zhang, 2016). In particular, human induced pluripotent stem cells (hiPSCs) can produce patient-specific neuronal cells and are useful in genome editing, allowing us to understand the pathogenic mechanisms associated with genetic risk factors (Lin et al., 2018; Kim et al., 2019). Despite the advantage of a relatively homogeneous production of high-purity neuronal cells, two-dimensional (2D) cultures, which differ from the human brain environment in terms of cell-to-cell or cell-to-matrix connections and spatial organization (Wang, 2018), have limited ability to replicate human brain development and pathogenesis.

Recently, it was reported that three-dimensional (3D) differentiation into the neuronal lineage from hPSCs could generate brain-like 3D tissue biomimetics, named brain organoids (Lancaster et al., 2013). Various types of brain organoids, such as brain region-specific organoids (Muguruma et al., 2015; Pasca et al., 2015; Sakaguchi et al., 2015; Jo et al., 2016; Qian et al., 2016) and assembloids (Bagley et al., 2017; Birey et al., 2017; Xiang et al., 2017), have been developed. These brain organoids replicate human-specific brain structures (Lancaster et al., 2013) and disease pathology (Jo et al., 2016; Burbulla et al., 2017), suggesting that brain organoids are more physiologically related to the human brain than animal models. Since brain organoids have no blood brain barrier and are difficult to reflect the systemic effects of drugs, brain organoids and animal models can become complementary human brain models. Brain organoid systems have modeled neurodevelopmental and neurodegenerative disorders, such as microcephaly, autism spectrum disorder, Alzheimer’s disease, and Parkinson’s disease (PD) (Lancaster et al., 2013; Mariani et al., 2015; Jo et al., 2016; Raja et al., 2016).

In this study, we aimed to develop simBOs, which could enable the rapid and robust production of uniform brain organoids for application in disease modeling and drug discovery. To shorten the period of differentiation and reduce the variability of brain organoids, we developed a step-wise differentiation method from hPSC-derived expandable primitive neural stem cells (pNSCs) (Li et al., 2011; Liu et al., 2012) to organoids. Transcriptome analysis was performed to establish the neuronal characteristics of simBOs and to understand the molecular differences between 2D and 3D environments in neuronal differentiation. Furthermore, we generated midbrain-like simBOs, which could replicate PD phenotype and drug response.

## Materials and Methods

### hiPSCs Maintenance and Differentiation Into pNSCs

Human-induced pluripotent stem cells (hiPSCs) were obtained from a healthy donor and generated from fibroblasts (AG02261, Coriell Cells Repositories, Camden, NJ, United States) as previously described (Lee M. et al., 2019) and PD-patient (*LRRK2*^G2019S^)-derived induced pluripotent stem cells (iPSCs) (Son et al., 2017) and gene-corrected iPSCs (Lee and Chung, 2019) were used for PD modeling. hiPSCs were maintained in TeSR-E8 medium (STEMCELL Technologies, Vancouver, Canada) and subcultured with ReLeSR (STEMCELL Technologies) according to the manufacturer’s instructions. For reprogramming purposes, human fibroblasts were exempted from IRB review by the Public Institutional Review Board designated by the Ministry of Health and Welfare (P01-201802-31-001).

Primitive neural stem cells (pNSCs) were induced and maintained as previously described (Liu et al., 2012). Briefly, hiPSCs were seeded onto Geltrex (Thermo Fisher Scientific, Waltham, MA, United States)-coated 35 mm dishes (Corning, NY, United States) for 20% confluence. The next day, the medium was switched to Neural Induction Medium-I [NIM-I: 50% Advanced DMEM/F12 (Gibco, Invitrogen, Waltham, MA, United States), 50% Neurobasal (Gibco), 1× N2 (Gibco), 1× B27 (Gibco), 1% Glutamax (Gibco), 0.5% Albumax (Thermo Fisher Scientific), and 10 ng/mL hLIF (PeproTech, Rocky Hill, NJ, United States) supplemented with 4 μM CHIR99021 (GSK3 inhibitor; Tocris Bioscience, Bristol, United Kingdom), 3 μM SB431542 (TGF-β receptor inhibitor; Tocris), 2 μM Dorsomorphin (Tocri), and 0.1 μM Compound E (Tocris)] for 2 days. The medium was then switched to NIM-II (NIM-I excluding Dorsomorphin) for another 5 days. The cells were transferred onto Geltrex-coated dishes with Accutase (MilliporeSigma, Billerica, MA, United States) and maintained in Neural Stem Cell Maintenance Medium (NSMM: 50% Advanced DMEM/F12, 50% Neurobasal, 1× N2, 1× B27, 1% Glutamax, 0.5% Albumax, 10 ng/mL hLIF, 3 μM CHIR99021, and 2 μM SB431542).

### Spontaneous Differentiation of pNSCs in 2D Culture Dishes

pNSCs were dissociated into single cells using Accutase. 80,000 cells were plated on Geltrex-coated 24- or 4-well tissue culture plates (Corning) and cultured in NSMM for 4 days. Then, the medium was switched to differentiation medium [DMEM/F12, 1× N2, 1× B27, 200 ng/mL dbcAMP (Enzo Life Sciences, Basel, Switzerland) and 5 μM DAPT (Tocris)] with 10 ng/mL BDNF (PeproTech), 10 ng/mL GDNF (PeproTech), and fed every other day for 10–20 days.

### Generation of Simplified Brain Organoids (simBOs)

To permit pNSCs self-organization, single-cell-dissociated pNSCs were seeded on wells of ultra-low attachment 96-well plates (Corning Incorporated, Kennebunk, ME, United States) and cultured in NSMM. 100,000 cells and 50,000 cells were used for self-organization of simBOs and midbrain-like simBOs, respectively. After 4 days, 96 spheres of pNSCwere transferred into a bioreactor (ABLE Corporation, Tokyo, Japan) and maintained in 5–10 ml of spontaneous differentiation medium (differentiation medium with 10 ng/mL BDNF and 10 ng/mL GDNF) or midbrain-specification medium [differentiation medium with 100 ng/mL Shh (R&D systems, Minneapolis, MN, United States) and 100 ng/mL FGF8 (PeproTech, TX, United States)] for 10–20 days. The culture medium was changed every 3 days. To check the efficacy of LRRK2 kinase inhibitors in PD patient-derived midbrain-like simBOs, 5 μM PFE-360 (MedChem Express, Sollentuna, Sweden) was used for 3 days.

### Size Measurement of simBOs

The size of simBOs was determined using phase contrast images by measuring the diameter of 10 to 25 organoids with the ImageJ software (National Institutes of Health, Bethesda, MD, United States). The area was calculated using the following equation:

$$\text{Area}=\left(\frac{\mathrm{major}\mathrm{axis}}{2}\right)\times \left(\frac{\mathrm{minor}\mathrm{axis}}{2}\right)\times \pi$$

### RNA Preparation, cDNA Synthesis, and Quantitative Real-Time PCR

Twelve organoids were lysed for each sample using Easyblue (iNtRON Biotechnology, Seongnam-si, South Korea) and total RNA was extracted according to the manufacturer’s instructions. cDNA was synthesized from 1 μg of total RNA using the iScript cDNA synthesis kit (Bio-Rad, Hercules, CA, United States). Quantitative real-time PCR was performed using a 1/50 concentration of the obtained cDNA with iQ SYBR Green Supermix (Bio-Rad) on an Applied Biosystems 7500 Fast Real-Time PCR instrument system (Thermo Fisher Scientific). The cycle threshold (*C*t) value for each target gene was determined using the software provided by the manufacturer. The expression data were normalized to the *C*t value of *RPL7* (Efe et al., 2011). The primer sequences used in this study are shown in Supplementary Table 1.

### Immunohistochemistry and Immunocytochemistry

Eight to 12 organoids of each sample were fixed with 4% paraformaldehyde (Electron Microscopy Sciences, Hatfield, PA, United States) and incubated overnight at 4°C. Fixed samples were incubated with sucrose solutions of an increasing gradient (10, 20, and 30%) prepared in Dulbecco’s phosphate-buffered saline (DPBS; WELGENE, Daegu, South Korea) for cryoprotection. Samples were embedded in optimal cutting temperature (O.C.T.) compound (Sakura Finetek, Tokyo, Japan) and frozen in liquid nitrogen. The organoids were sectioned using a cryostat microtome into 10–15 μm-thick sections. The cells cultured in 2D were grown in 24- or 4-well tissue culture plates and fixed with 4% paraformaldehyde and 0.15% picric acid (Sigma-Aldrich, St. Louis, MO, United States) in DPBS for 15 min.

For immunostaining, samples were blocked and permeabilized with 3% bovine serum albumin (BSA; Thermo Fisher Scientific) and 0.3% Triton X-100 (Sigma-Aldrich) in DPBS for 1 h at room temperature, as previously described (Lee et al., 2020). All primary antibodies were diluted in 1% BSA and incubated overnight at 4°C. After iterative washing with 0.1% BSA in DPBS, the samples were incubated with Alexa-594- or Alexa-488-conjugated secondary antibodies (Thermo Fisher Scientific) for 1 h at room temperature. All fluorescent images were acquired using an Evos FL auto 2 imaging system (Thermo Fisher Scientific). The antibodies used in this study are listed in Supplementary Table 2.

### Microarray Analysis

For comprehensive gene expression analysis, 12 organoids were used for each sample and cDNA microarray was performed at e-biogen (ebiogen, Seoul, South Korea) using an Agilent Human GE 4 × 44K v2 Microarray Kit (Agilent, Santa Clara, CA, United States), as described previously (Lee M.O. et al., 2019). Data normalization was performed with GeneSpring software (Agilent) and differentially expressed genes were sorted by fold-change of gene expression and heat map was presented using the Multi Experiment Viewer software (MeV version 4.9.0^1^). Gene set enrichment analysis was performed with GSEA (GSEA^2^) (Mootha et al., 2003; Subramanian et al., 2005) and pathway enrichment in the Reactome Database^3^ was performed using Cytoscape software^4^.

### Western Blotting

Whole-cell extracts of 8 to 12 organoids were prepared using RIPA buffer (Sigma-Aldrich) containing 1 mM PMSF (Sigma-Aldrich), a cocktail of protease inhibitors, and 1× phosSTOPTM (Roche Applied Science, Basel, Switzerland). Protein concentrations were determined using a Pierce BCA protein assay kit (Thermo Fisher Scientific). Equal amounts of total protein were separated on MP TGX Precast Gels (Bio-Rad) and transferred to polyvinylidene fluoride membranes (Bio-Rad). The membranes were blocked in Tris-buffered saline (LPS Solution, Daejeon, South Korea) containing 0.05% Tween-20 (TBST; Sigma-Aldrich) and 1.5% BSA for 1 h at room temperature and then incubated with specific primary antibodies overnight at 4°C. After washing with TBST, the samples were incubated for 1 h at room temperature with horseradish peroxidase-conjugated secondary antibodies (Cell Signaling Technology, Danvers, MA, United States; 1:5000). The antibodies used in this study are listed in Supplementary Table 2. The blots were developed using ECL Select Western Blotting Detection Reagent (GE Healthcare, Little Chalfont, United Kingdom) and band images were acquired using the luminescent image analyzer LAS-3000 (Fuji Photo Film GMBH, Tokyo, Japan). The band intensity was quantified using ImageJ software.

### Dopamine Secretion

To determine the amount of dopamine released from simBOs, 24 h-culture medium was harvested from 24-well containing 8 organoids at 13 days post-differentiation and subjected to enzyme-linked immunoassay with the Dopamine Research ELISA kit (LDN, Nordhorn, Germany) according to the manufacturer’s instructions. In brief, dopamine was extracted, acylated, and enzymatically converted. Once the antibody bound to the bottom of the plates captured the converted dopamine, the first was detected using an anti-rabbit IgG-peroxidase conjugate with the substrates. Samples were quantified by comparing their absorbance values with a standard curve prepared with known standard concentrations.

### Statistical Analysis

**FIGURE 1:**
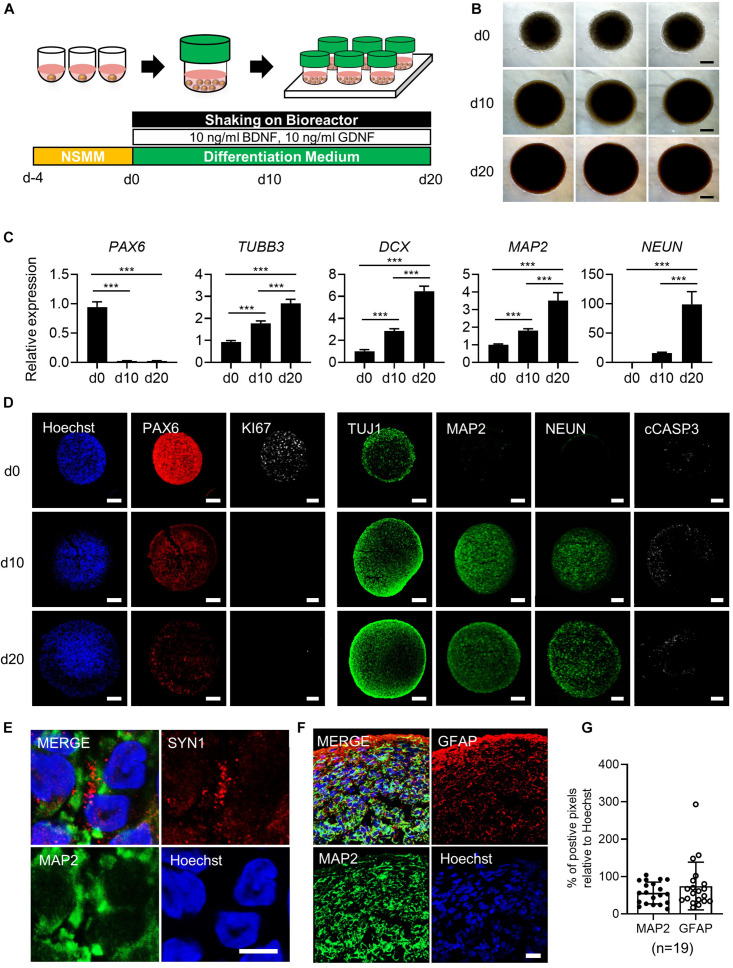
Generation of simplified brain organoids (simBOs) from human primitive neural stem cells (pNSC). **(A)** Graphical schematics for the generation of pNSCs into simBOs. **(B)** Phase contrast images of simBOs at days 0, 10, and 20 of spontaneous differentiation. Scale bars represent 200 μm. **(C)** Quantitative reverse transcription polymerase chain reaction (qRT-PCR) analysis for neural progenitor (*PAX6*) and neuronal (*TUBB3*, *DCX*, *MAP2*, and, *NEUN*) markers in simBOs at days 0, 10, and 20 of spontaneous differentiation. Expression value was normalized to those of day 0 (d0). Data represent mean ± standard error (SE) from three technical replicates. Statistical significance of Turkey’s multiple comparisons ***p < 0.001 after one-way ANOVA. **(D)** Immunofluorescence analysis of simBOs with proliferating neural progenitor markers (PAX6 and Ki67), neuronal markers (TUJ1, MAP2, and NEUN), and apoptotic cell marker (cleaved caspase III; cCASP3) at indicated times. Scale bars represent 100 μm. **(E)** Immunofluorescence analysis of simBOs with a mature neuronal marker (MAP2) and a synaptic marker (SYN1) in simBOs after day 10 of spontaneous differentiation. Scale bar represents 5 μm. **(F)** Immunofluorescence analysis of simBOs with mature neuronal marker (MAP2) and astroglial cell marker (GFAP) after day 10 of spontaneous differentiation. Scale bar represents 20 μm. **(G)** Quantitative graph of the percentage (%) of positive pixels of each marker relative to Hoechst. Data represent mean ± standard error (SE) (*n* = 19). No significant differences were observed.

Statistical significance was calculated with the unpaired *t*-test or analysis of variance (ANOVA) in GraphPad Prism version 8 for windows (GraphPad Software, Inc., CA, United States). Bar-charts (in Figures 1C, 3C, 5C, 6B, 7C,G,H) are presented as means ± standard error (SE) and the box-and-whisker plots in Figures 1G, 7I are presented as min to max with all points. Size similarity of simBOs (Supplementary Figures 2B, S3B, S6A,B) was presented by calculating the standard deviation/average. Experimental replicates of each experiment was indicated in the figure legend.

## Results

### Generation of simBOs From hPSC-Derived pNSCs

Given that pNSCs differentiated from hPSCs are highly proliferative, cryopreservable, and capable of producing various cell types present in the brain and nervous system (Li et al., 2011; Liu et al., 2012), we hypothesized that self-organization of pNSCs and subsequent differentiation in a stirred bioreactor could rapidly generate simBOs. To address this idea, we differentiated pNSCs from hiPSCs derived from healthy human somatic cells following a previously reported procedure (Supplementary Figures 1A,B) (Liu et al., 2012). pNSCs showed high expression of neuronal stem cell markers (PAX6, NESTIN, and SOX2), marginal expression of pan-neuronal marker (TUJ1), and no expression of mature neuronal marker (MAP2), implying that pNSCs harboring the characteristics of neural stem cells (Supplementary Figure 1C).

To generate simBOs, pNSCs were self-organized in ultra-low attachment 96-well plates with the indicated number of cells (30,000 to 200,000 cells per organoid). Four days later, the cell aggregates were transferred to a bioreactor and differentiated in spontaneous differentiation medium for 10–20 days (Figure 1A). To analyze morphological homogeneity and growth, the size of each organoid was measured on days 0, 10, and 20 (Figure 1B and Supplementary Figures 2A,B). Up to a seeding density of 100,000 cells per organoid, simBOs were homogeneously produced and showed less than 2% of standard deviation/average (STDEV) of spheroid size. However, at a seeding density of 200,000 cells, STDEV was over 2–7% (Supplementary Figures 2A,B). Additionally, this seeding density significantly impaired the induction of mature neuronal marker (*NEUN*) (Supplementary Figure 2C) and facilitated apoptosis (Supplementary Figure 2D). Thus, we set up a protocol with an initial number of 100,000 cells for the self-organization of pNSCs.

To verify the robust reproducibility of the system, we produced up to 96 simBOs at a time, and these were cultured in one bottle with 5–10 mL of differentiation medium. Since the capacity of the bottle was of 30 mL, increasing the amount of medium is expected to increase the number of simBOs that can be cultured in a bottle. The bioreactor can accommodate up to six bottles, suggesting that at least are 576 simBOs (minimum) can be produced and cultured at each time (Supplementary Figure 3A). Importantly, given that the pNSCs used as starting cells for organoid production are highly proliferative and stable, we were able to culture a large number of simBOs with the established bioreactor system. Analysis of three independent batches of simBOs production confirmed very similar size distribution within each batch (STDEV/AVG < 5%) (Supplementary Figures 3B,C).

To characterize simBOs, we first examined the expression of neuronal markers in the levels of transcripts and proteins to evaluate time-dependent neuronal differentiation. Quantitative real-time PCR (qRT-PCR) analysis revealed reduced expression of the neural progenitor marker gene (*PAX6*) and enhanced expression of neuronal marker genes (*TUBB3*, *DCX*, *MAP2*, and *NEUN*) during spontaneous differentiation until day 20 (Figure 1C). Homogenous expression of neuronal markers (*PAX6*, *DCX*, *MAP2*, *and NEUN*) was showed in distinct batches of simBOs (Supplementary Figure 4). Consistently, immunofluorescence analysis also showed a decrease in neural stem cell marker (PAX6) and an increase in neuronal markers (TUJ1, MAP2, and NEUN) with a homogeneous pattern between organoids through differentiation (Figure 1D and Supplementary Figures 5A,B). In particular, the proliferation marker (Ki67) was substantially reduced after differentiation, implying differentiation into post-mitotic neurons. Moreover, the staining of cell death marker (cCASP3) indicated no significant formation of apoptotic bodies in simBOs (Figure 1D). We also observed the expression of a synaptic marker (SYN1), suggesting that our simBOs contained functionally mature neurons (Figure 1E). Next, we analyzed the distribution of neuron and astroglia in simBOs. MAP2^+^ neuronal cells were evenly distributed throughout the organoids, whereas GFAP^+^ astroglial cells were enriched at the edges of organoids, and were also observed around neurons on day 10 simBOs (Figure 1F). Comparing the content of the two cells through pixel analysis of immunostained images, GFAP^+^ astroglial cells were distributed in a similar amount of MAP2^+^ neurons. To confirm the reproducible production of simBOs from various pNSC lines, we generated simBOs from two independent pNSCs [Parkinson disease (PD)-patient derived LRRK2G2019S pNSCs and gene-corrected control pNSCs] (Supplementary Figure 6). The result demonstrated that simBOs can be uniformly produced form a variety of human pNSCs. Taken together, these data demonstrated that simBOs are rapidly and homogenously produced brain organoids, which are structurally simple but uniform.

### Molecular Characteristics of the simBOs

**FIGURE 2:**
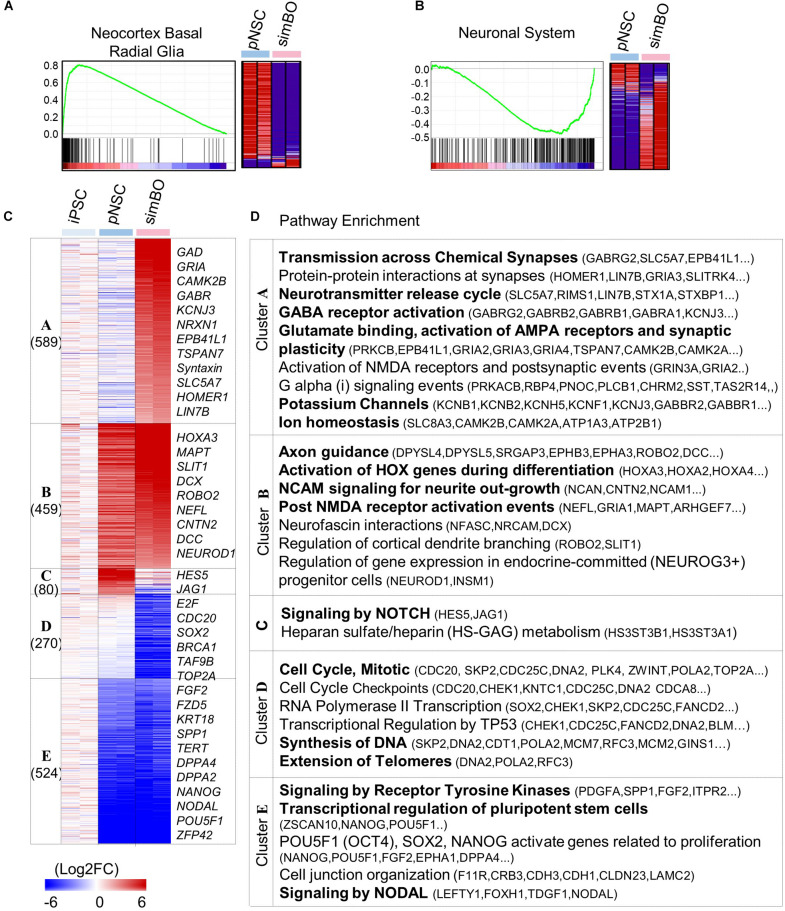
Transcriptomic analysis of simBOs. **(A,B)** Enrichment plot and Heat map for the molecular signature of Neocortex Basal radial glia **(A)** and neuronal system **(B)** analyzing by gene set enrichment analysis of pNSC and simBOs. **(C)** Heat map of differentially expressed genes among iPSCs, pNSCs, and simBOs. Expression value was normalized to those of iPSCs. Cluster name and gene number in each cluster were indicated in left panel and representative gene names in each cluster were indicated in the right panel. Color scale of expression value were presented in lower panel. **(D)** Chart describing representative enrichment reactome pathways and entities related to differentially expressed genes in each cluster.

To access the molecular characteristics of simBOs, we performed a comprehensive gene expression analysis. Gene set enrichment analysis (GSEA) between pNSCs and simBOs indicated that pNSCs have a molecular signature of neocortex basal radial glia (Figure 2A) and simBOs of the neuronal system (Figure 2B). To further analyze transcriptional changes during the step-wise differentiation (hiPSCs-pNSCs-simBOs), differentially expressed genes were selected by a fourfold or higher change and clustered into five groups (clusters A–E) (Figures 2C,D). The 524 genes in cluster E were decreased in both pNSCs and simBOs compared to hiPSCs, which were involved in the transcriptional regulation of pluripotent stem cells, signaling by nodal, and signaling by receptor tyrosine kinases. On the other hand, the 270 genes in cluster D, associated with cell cycle, DNA synthesis, and telomere extension, were only decreased in simBOs, implying that iPSCs and pNSCs consisted of proliferative cells, but simBOs were post-mitotic.

The pNSC-specific genes were grouped in cluster C, involving the signaling by NOTCH and heparin sulfate/heparin metabolism, and the 459 genes (in cluster B) related to neuronal development in terms of axon guidance, activation of HOX genes during differentiation, and NCAM signaling for neurite out-growth, were increased in both pNSCs and simBOs. In contrast, the 589 genes in cluster A related to neuronal functionality (e.g., transmission across chemical synapses, neurotransmitter release cycle, GABA receptor activation, glutamate binding, potassium channels, and ion homeostasis) were increased only in simBOs. These data suggest that pNSCs have the properties of NOTCH signaling-dependent proliferative neural stem cells and 3D differentiation induces rapid conversion into post-mitotic functional neurons.

### Enhanced Neuronal Differentiation in the simBOs

**FIGURE 3:**
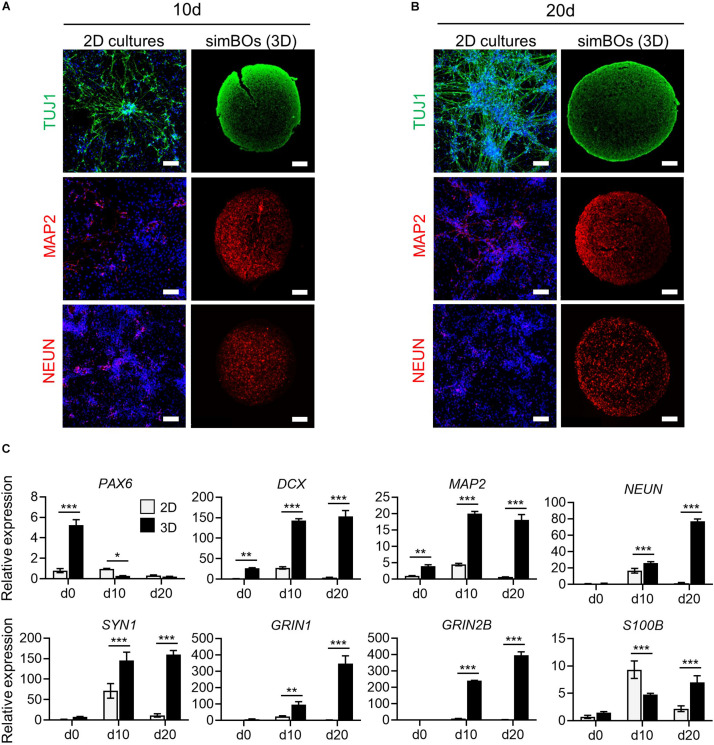
Comparison of neuronal differentiation potential in 2D vs. 3D. **(A,B)** Immunofluorescence analysis to compare neuronal differentiation potential between 2D and 3D with neuronal markers (TUJ1, MAP2, and, NEUN) in simBOs at day 10 **(A)** and 20 **(B)** of spontaneous differentiation. Scale bars represent 100 μm. **(C)** Quantitative reverse transcription polymerase chain reaction (qRT-PCR) analysis for neural progenitor marker (*PAX6*), pan-neuronal markers (*DCX*, *MAP2*, *NEUN*, and *SYN1*), glutamatergic neuronal marker (*GRIN1*), GABAergic neuronal marker (*GRIN2B*) and glial marker (*S100B*) in simplified brain organoids (simBOs; 3D) and 2D differentiation samples. Expression value was normalized to those of human primitive neural stem cells (pNSC) in 2D (d0). Data represent mean ± standard error (SE) from three technical replicates. Statistical significance of Sidak’s multiple comparisons *p < 0.05, **p < 0.01, ***p < 0.001 after two-way ANOVA.

Next, we assessed whether a 3D environment could promote enhanced neuronal differentiation of simBOs. To directly compare the neuronal differentiation pattern between 2D cultures and simBOs, pNSCs were differentiated in a 2D environment with the same differentiation medium used for simBOs. Immunofluorescence analysis showed that neurons differentiated in 2D cultures had higher level of TUJ1, a neurofilament protein, but lower expression of mature neuronal markers (MAP2 and NEUN) than simBOs (Figures 3A,B). Consistent with this result, rapid reduction of the neural stem cell marker (*PAX6*) and robust induction of immature (*DCX*) and mature (*MAP2*, *NEUN*, and *SYN1*) neuronal markers in the simBOs was confirmed via qRT-PCR (Figure 3C). Moreover, *GRIN1* and *GRIN2B*, which play a crucial role in excitatory glutamatergic and inhibitory GABAergic synapses, respectively, also dramatically increased in the simBOs. However, *S100B*, the glial-specific protein, showed no difference between 2D cultures and simBOs, suggesting that the 3D environment could trigger neuron-specific maturation within 10 days.

**FIGURE 4:**
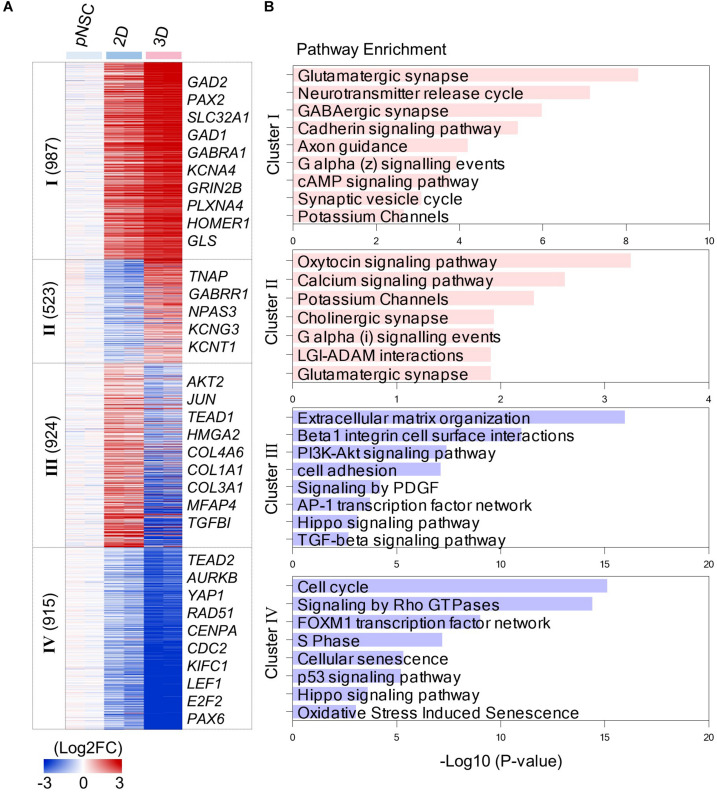
Transcriptomic analysis of simBOs and 2D differentiation. **(A)** Heat map of differentially expressed genes between 2D and 3D differentiation. Expression value was normalized to those of iPSCs. Cluster name and gene number in each cluster were indicated in left panel and representative gene names in each cluster were indicated in the right panel. Color scale of expression value were presented in lower panel. **(B)** Bar chart for the –Log10(*p*.value) of representative enriched pathway calculated by reactome pathway enrichment analysis.

A comparison of transcriptome data between the differentiation in 2D and 3D environments showed that there were 1,490 and 1,839 genes enriched in simBOs (clusters I and II) and 2D (clusters III and IV) cultures, respectively (Figure 4A). Gene ontology analysis according to cellular components showed that both environments markedly increased the expression of genes encoding proteins that act at different intracellular locations (Supplementary Figure 7). The simBOs were promoted genes encoding proteins of the cellular component related to neuronal functionality such as neuron projection, synapse, axon, dendritic spine, voltage-gated potassium, or calcium channel complex (clusters I and II). On the other hand, the 2D cultures were enhanced the expression of genes related to the extracellular space, elastic fiber, midbody, and kinetochore (clusters III and IV).

By analyzing the enriched pathway of 4 cluster gene sets, we found that simBOs were improved neuronal characteristics and the extracellular environment with higher similarity to the human brain (Figure 4B). In detail, cluster I consisted of 987 genes that increased in 2D cultures and further increased in simBOs. These differentially expressed genes corresponded to a representative enrichment in the pathways associated with differentiation into functional neurons such as glutamatergic synapses, GABAergic synapses, neurotransmitter release cycles, axon guidance, and potassium channels. Furthermore, the 459 genes in cluster II were only increased in simBOs and partly included genes belonging to pathways of calcium signaling, potassium channels, cholinergic synapses, and glutamatergic synapses (Figure 4B and Supplementary Figure 8).

In contrast, the 2D cultures were increased the expression of 924 genes (cluster III) involved in the extracellular matrix (ECM) organization and focal adhesion, whereas these genes were decreased in simBOs (Figure 4B and Supplementary Figure 9). Fibrous proteins such as collagen and fibronectin are barely present in the extracellular space of the brain, which gives the brain its soft properties (Hong and Do, 2019). To verify whether simBOs created an ECM environment closer to the *in vivo* brain, we compared 74 differentially expressed genes related to ECM organization during *in vitro* neural differentiation or *in vivo* human brain development. As shown in the Supplementary Figure 10, simBOs showed a similar expression pattern of ECM-related genes to that of the *in vivo* brain development, with reduced number of fibrous ECM organization genes. In addition, the cluster IV genes, which were reduced in the 2D cultures and further decreased in the simBOs, are involved in cell cycles, signaling by rho GTPase, and S phase (Figure 4B and Supplementary Figure 9). Interestingly, genes involved in the Hippo signaling pathway were enriched in clusters III and IV (Figure 4B), implying a possible role in differentiation of simBO. Taken together, these results suggest that the 3D environment during neuronal differentiation enhanced differentiation into post-mitotic mature neurons and brain-like ECM.

### Cytoplasmic Translocation of YAP1 in the 3D Environment

**FIGURE 5:**
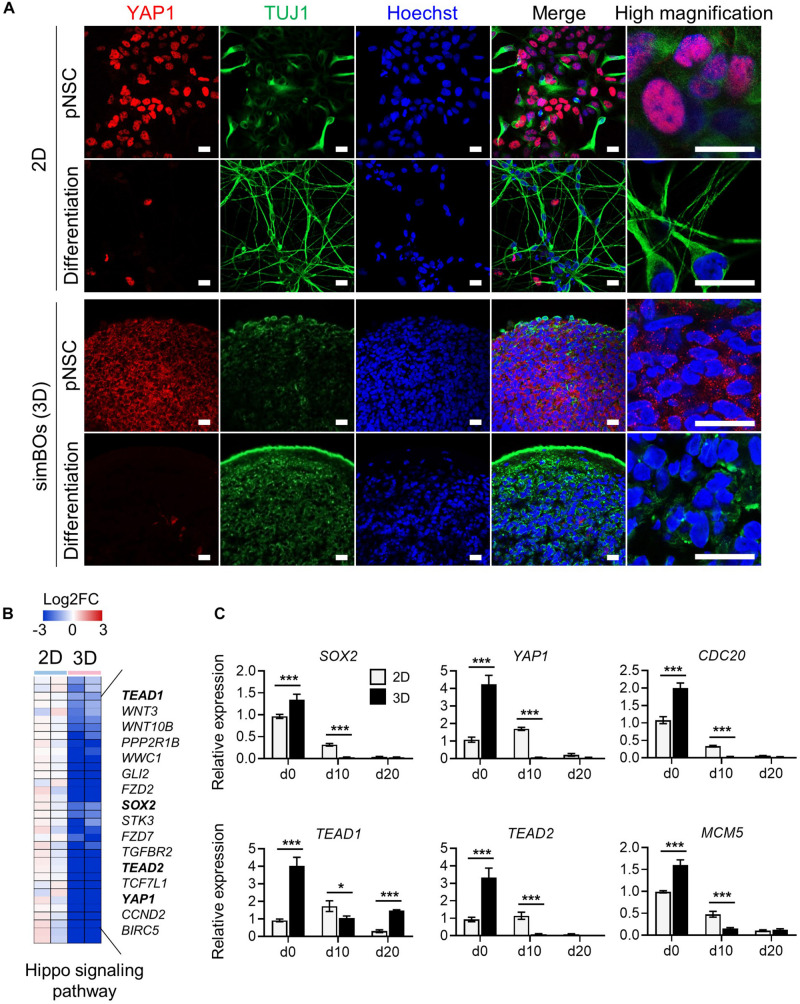
Subcellular localization and transcription regulation of YAP1. **(A)** Subcellular localization of YAP1 and TUJ1. Scale bars represent 20 μm. **(B)** Heat map for the Hippo signaling pathway genes differentially expressed during 2D and 3D differentiation. Representative gene names were presented in right panel and color scale was presented upper panel. **(C)** Quantitative reverse transcription polymerase chain reaction (qRT-PCR) analysis for *YAP1* and direct targets of YAP1 (*SOX2*, *TEAD1*, *TEAD2*, *CDC20*, and *MCM5*) in simplified brain organoids (simBOs; 3D) and 2D differentiation samples. Expression value was normalized to those of human primitive neural stem cells (pNSCs) in 2D (d0). Data represent mean ± standard error (SE) from three technical replicates. Statistical significance of Sidak’s multiple comparisons *p < 0.05, ***p < 0.001 after two-way ANOVA.

Next, we explored how the 3D environment can enhance rapid neuronal differentiation in the same differentiation medium. The 3D environment provides a relatively low-stiffness matrix closer to that of tissues than the plastic surface of culture dishes (Baker and Chen, 2012) and Yes-associated protein 1 (YAP1), a transcription co-activator of the Hippo signaling pathway, is a well-known mechanosensor that modulates cellular behavior through cytoplasmic-nuclear translocation by responding to stimuli from the mechanical environment (Dupont et al., 2011). Moreover, YAP1 promotes the proliferation of neural stem cells and inhibits neuronal differentiation (Cao et al., 2008; Zhang et al., 2012). Thus, we hypothesized that low stiffness in a 3D environment could translocate YAP1 proteins into the cytoplasm, and this could promote neuronal differentiation by inhibiting the function of YAP1 as a transcriptional co-activator. To test this hypothesis, we examined the subcellular localization of YAP1 in the pNSCs and differentiated neuronal cells depending on the 2D or 3D environment. As expected, Figure 5A shows that YAP1 is mainly located in the nucleus in the 2D environment, whereas it is detected in the cytoplasm in the simBOs, suggesting functional impairment of YAP1. In addition, neuronal differentiation rapidly diminished YAP1 expression in differentiated neurons (Figure 5A).

Furthermore, transcriptome data showed that 3D differentiation further decreased genes related to the Hippo signaling pathway including *YAP1*, *TEAD1*, and *TEAD2*, and transcription factors directly bound to YAP1 and mediated YAP1-dependent transcription regulations (Cao et al., 2008) (Figure 5B). To confirm the expression of *YAP1* and its targets, we performed RT-PCR in the pNSCs and differentiated cells in the 2D or 3D environment (Figure 5C). *YAP1*, *TEAD1*, *and TEAD2* expression decreased more rapidly during differentiation in the 3D environment, comparably to the expression pattern of the neural stem cell marker *SOX2*. Moreover, *CDC20* and *MCM5*, which are direct targets of the YAP1-TEADs complex and regulators of the cell cycle, also had their expression decreased more rapidly during differentiation in the 3D environment compared to the 2D environment. These data suggest that the rapid differentiation of simBOs is related to the dysfunction of YAP1 by translocation.

### Generation of Midbrain-Like simBOs and PD Modeling

**FIGURE 6:**
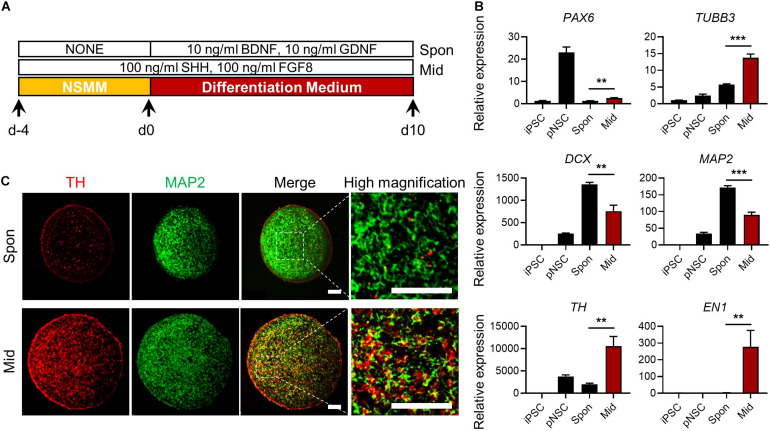
Generation of midbrain-like simplified brain organoids (simBOs). **(A)** Graphical schematics for midbrain-like simBOs. **(B)** Quantitative reverse transcription polymerase chain reaction (qRT-PCR) analysis for neural progenitor marker (*PAX6*), pan-neuronal markers (*TUBB3*, *DCX*, and *MAP2*) and dopaminergic neuron markers (*TH* and *EN-1*) in induced pluripotent stem cells (iPSCs), human primitive neural stem cells (pNSCs), spontaneously differentiated-simBOs (Spon), and midbrain-like simBOs (Mid). Expression value was normalized to those of iPSCs. Data represent mean ± standard error (SE) from three technical replicates. Statistical significance of **p < 0.01, ***p < 0.001 was calculated by unpaired *t*-test. **(C)** Immunofluorescence analysis with pan-neuronal markers (MAP2) and dopaminergic neuronal marker (TH) in Spon-simBOs and Mid-simBOs. Scale bars represent 100 μm.

Next, we examined whether simBOs could be specified into midbrain-like organoids, which can replicate the pathology and drug response of PD. Because sonic hedgehog (Shh) and fibroblast growth factor 8 (FGF8) are key signaling molecules for midbrain specification (Arenas et al., 2015), we treated simBOs with Shh and FGF8 during differentiation (Figure 6A). Compared to spontaneously differentiated simBOs, the dopaminergic neuronal markers (*TH* and *EN1*) were robustly up-regulated in the midbrain-like simBOs (Figure 6B). In contrast, neuronal markers (*TUBB3*, *DCX*, and *MAP2*) were highly expressed in both spontaneously differentiated simBOs and midbrain-like simBOs. Immunofluorescence staining showed that TH^+^(tyrosine hydroxylase)/MAP2^+^ dopaminergic neurons were distributed throughout the midbrain-like simBOs (Figure 6C).

**FIGURE 7:**
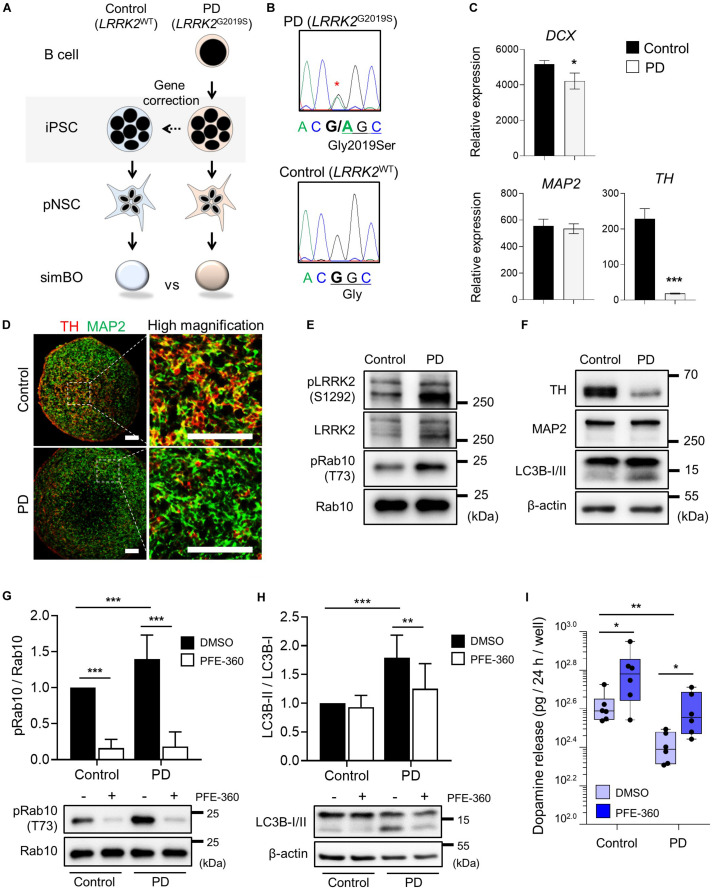
Parkinson’s modeling with midbrain-like simplified brain organoids (simBOs). **(A)** Schematic flow diagram to depict the establishment of PD patient-derived simBOs from harboring *LRRK2* G2019S mutation (PD) and gene-corrected control iPSCs (Control). **(B)** DNA sequencing data for the *LRRK2* G2019S (Gly to Ser) mutation and correction of this gene in the iPSCs. **(C)** Quantitative reverse transcription polymerase chain reaction (qRT-PCR) analysis for pan-neuronal markers (DCX and MAP2) and dopaminergic neuronal marker (TH). Expression value was normalized to those of induced pluripotent stem cells (iPSCs). Data represent mean standard error (SE) from three technical replicates. Statistical significance of *p < 0.05, ***p < 0.001 was calculated by unpaired *t*-test. **(D)** Immunofluorescence analysis with pan-neuronal marker (MAP2) and dopaminergic neuronal marker (TH). Scale bars represent 100 μm. **(E,F)** Western blotting for indicated proteins in PD-simBOs. β-actin was used as loading control. **(G,H)** Representative western blotting and quantitative graph for LC3B-II normalized to LC3B-I. **(G)** and pRab10 normalized to total Rab10 **(H)** at day 13 with or without LRRK2 kinase inhibitor (PFE-360) treatment for 3 days. β-actin was used as loading control. Data represent mean ± SE from three batches, two technical replicates. Statistical significance of Turkey’s multiple comparisons **p < 0.01, ***p < 0.001 after two-way ANOVA. **(I)** Dopamine secretion of simBOs was measured in the presence of DMSO or PFE-360. For analysis, cultured media for 24-h were obtained from the wells containing eight simBOs each. Data represent mean ± SE from two batches, three technical replicates. Statistical significance of *p < 0.05, **p < 0.01 was calculated by unpaired *t*-test.

For PD modeling, we used PD patient-derived iPSCs harboring *LRRK2*^G2019S^ point mutation (PD-iPSCs) and gene-corrected control iPSCs (Control-iPSCs) by BAC-based homologous recombination (Son et al., 2017; Lee and Chung, 2019) (Figure 7A). We performed DNA sequencing and confirmed a heterozygous *LRRK2*^G2019S^ mutation in PD-iPSCs and corrected this mutation in control-iPSCs (Figure 7B). These iPSCs were fully characterized in the context of alkaline phosphatase activity, expression of pluripotency markers (OCT-3/4 and NANOG) (Supplementary Figure 11A), and embryonic body formation (Supplementary Figure 11B). Additionally, both iPSCs showed a normal karyotype (Supplementary Figure 11C) and the same genotype on short tandem repeat (STR), proving to be isotype-paired cells from an identical person (Supplementary Figure 11D). Finally, we obtained expandable pNSCs from these iPSCs and characterized them by immunofluorescence analysis of neural markers (NESTIN) and neurofilament protein (TUJ1) (Supplementary Figure 11E).

### Generation of PD Patient-Derived (*LRRK2*^G2019S^) and Gene-Corrected Control Midbrain-Like simBOs

Next, we generated midbrain-like simBOs from PD patient-derived (*LRRK2*^G2019S^)-pNSCs (PD-simBOs) and gene-corrected control-pNSCs (Control-simBOs). These midbrain-like simBOs showed no differences in the expression of neuronal marker genes (*DCX* and *MAP2*), but the mDA neuronal marker gene (*TH*) expression was substantially decreased in PD-simBOs (Figure 7C). Consistently, we observed that dopaminergic neurons (TH^+^/MAP2^+^) were decreased in PD-simBOs compared to control-simBOs (Figure 7D). Moreover, to confirm LRRK2 kinase activity in these midbrain-like simBOs, we examined phosphorylation sites of LRRK2 kinase substrates, Ser1292 in LRRK2 (Sheng et al., 2012) and Thr73 in Rab10 (Thirstrup et al., 2017). The phosphorylation of LRRK2 kinase substrates was increased in PD-simBOs compared to control-simBOs (Figure 7E), demonstrating that PD-simBOs replicated the molecular signatures of familial PD. Consistently with previous results, MAP2 expression was quite similar between PD- and control-simBOs, but TH was decreased in PD-simBOs (Figure 7F). Additionally, the levels of LC3B-II, an autophagy marker, were increased in PD-simBOs (Figure 7F). Previously, it was reported that the *LRRK2*^G2019S^ mutation caused autophagy, which resulted in neurite autophagy and shortening (Plowey et al., 2008; Bravo-San Pedro et al., 2013). Thus, we hypothesized that the elevated autophagy in PD-simBOs could be rescued by suppression of LRRK2 kinase activity. When we treated PD models of midbrain-like simBOs with LRRK2 kinase inhibitor, PFE-360, Rab10 phosphorylation was significantly suppressed in both PD- and control-simBOs (Figure 7G) and the LC3B-II levels were rescued in PD-simBOs (Figure 7H), suggesting autophagy are regulated by LRRK2 kinase activity in PD-simBOs.

Furthermore, we demonstrated that reduced dopamine release in PD-simBOs could be rescued by treatment with PFE-360 (Figure 7I), implying functional improvement of dopaminergic neurons by the LRRK2 inhibitor. These data indicate that midbrain-like simBOs from PD-iPSC-derived pNSCs could replicate the phenotype of PD and drug response.

## Discussion

Brain organoids are a breakthrough technology for studying embryonic development and disease pathology in the human brain. In particular, brain organoid models replicating various brain disorders are expected to be used as a model for drug screening (Wang, 2018). However, the heterogeneity between organoids and the need for long-term differentiation hinders their further application. Recently, several brain organoids with homogeneous properties in cellular composition have been reported (Velasco et al., 2019; Yoon et al., 2019; Kwak et al., 2020; Sivitilli et al., 2020), but these brain organoids still require long-term differentiation because they use iPSCs as a starting material. In this study, we generated homogeneous simBOs in 2 weeks using expandable pNSCs as a starting material.

Consistently with our results, Nickels et al. (2020) have shown that the use of more committed cells as a starting material not only induces homogeneous properties between organoids but also shortens the differentiation period. The neuronal layer formation seen in cerebral organoids was not observed in simBOs, but they consisted of diverse types of neurons and astrocytes. This approach allowed us to obtain structurally simple, but fast and robustly producible simBOs. Moreover, we demonstrated that pNSCs could be also specified into midbrain-like simBOs for PD modeling and established a pair of isogenic simBOs with an *LRRK2*^G2019S^ point mutation replicating the pathology of familial PD. We found that PD-simBOs showed elevated LRRK2 kinase activity and reduction of TH^+^ dopaminergic neurons, while these disease phenotypes were rescued in the gene-corrected isogenic control simBOs, indicating that our simBOs are useful models for studying the pathogenesis of familial PD harboring an *LRRK2*^G2019S^ mutation and discovering novel therapeutic targets for this disease.

After the generation of midbrain organoids (Jo et al., 2016; Qian et al., 2016), various organoid models that replicate familial, sporadic, and toxin-induced PD have been developed (Kim et al., 2019; Smits et al., 2019; Chlebanowska et al., 2020; Kwak et al., 2020). These brain organoids may be suitable models, successfully replicating the midbrain-specific phenotypes, for example, neuromelanin granules or midbrain-specific electrophysiology, and the pathological signatures of PD, for example, aggregation of alpha-synuclein or mDA neuron-specific cell death. However, they still have limitations in terms of time and cost to be used for drug discovery based on high-throughput screening (HTS). Our midbrain-like simBOs are not an advanced midbrain-like organoid model, but they are a more suitable model for HTS, as we were able to achieve rapid and robust production of homogeneous organoids. Moreover, we observed that elevated PD phenotypes were rescued by treatment with the LRRK2 kinase inhibitor, demonstrating that our simBOs are a ready-to-use model for screening drug candidates for PD.

Interestingly, we found enhanced neuronal maturity in simBOs, compared to that in 2D differentiation. It had been previously shown that cellular and extracellular signatures of human brain tissue are promoted in 3D culture environment (Simao et al., 2018). Consistently with these data, our transcriptome data showed that the 3D environment decreased fibrous ECM in the extracellular space and increased synapse and membrane ion-channel complex formation. Brain tissues have a unique composition of ECM compared to other organs: they have relatively low levels of fibrous proteins such as collagen and high levels of glycoproteins and proteoglycans, corresponding to the soft properties of the brain (Hong and Do, 2019). Thus, the decreased fibrous ECM organization in simBOs confers them more similar characteristics to those of the *in vivo* brain. The ECM serves not only as an essential structural scaffold but also provides significant biomechanical and biochemical stimulations for morphogenesis, cellular differentiation, and tissue homeostasis (Frantz et al., 2010). In particular, ECM influences brain development, homeostasis, and disease (Barnes et al., 2017). Thus, the dysregulation of ECM shown in 2D environment may act as a drawback in neuronal differentiation and disease modeling. In addition, enhanced neuronal maturation in simBOs could be associated with the improved ECM environment.

The differentiation and behavior of cells are stiffly regulated not only by biochemical signals (chemical composition and ligand density) but also by biophysical cues (matrix stiffness, topography, and geometry) (Yao et al., 2013; Bao et al., 2019). We observed that the 3D environment differentiates pNSCs faster into mature neurons under the same medium composition. Analysis of differentially expressed genes between samples differentiated in 2D and 3D environments revealed that the transcriptional co-activator YAP1, a mechanosensitive protein, was robustly and more rapidly downregulated during 3D differentiation, similar to the expression pattern of the neural stem cell marker SOX2. These results are consistent with a previous report indicating that SOX2 is a direct transcription regulator of YAP1 (Seo et al., 2013). Previous studies have shown that YAP1 enhances the proliferation of neural progenitors and negatively regulates neuronal differentiation (Cao et al., 2008; Zhang et al., 2012; Chighizola et al., 2019). During neuronal differentiation, YAP1 is excluded from nuclei and undergoes proteasomal degradation (Zaltsman et al., 2019). Interestingly, the subcellular localization of YAP1 was already promoted in our simBOs before neuronal differentiation, suggesting that 3D environment promoted translocation of YAP1. Given that different extracellular features were observed between 2D and 3D environments (Simao et al., 2018) and the stiffness of 3D environment affect the localization and expression of YAP1 (Molina et al., 2019), we could postulate that the altered extracellular features in the 3D environment might promote neuronal differentiation via suppression of YAP1. Although it still remains elusive as to which 3D culture environmental cues modulate the expression and localization of YAP1, our data support that 3D models more closely replicate the physiological relevance of the *in vivo* brain.

## Conclusion

Our results demonstrate that simBOs present the minimum-sufficient specification for the brain organoid model for HTS and can be a useful platform for modeling various brain disease.

## Data Availability Statement

The datasets generated for this study can be found in Gene Expression Omnibus (GSE156396).

## Ethics Statement

The studies involving human participants were reviewed and approved by the Public Institutional Review Board designated by the Ministry of Health and Welfare (P01-201802-31-001). The patients/participants provided their written informed consent to participate in this study.

## Author Contributions

JH, M-OL, and JK: conceptualization. AB, ML, and S-KC: validation. JSK and SK: formal analysis. JH and M-OL: writing—original draft preparation. M-OL and JK: writing—review and editing. All authors have read and agreed with the published version of the manuscript.

## Conflict of Interest

The authors declare that the research was conducted in the absence of any commercial or financial relationships that could be construed as a potential conflict of interest.

## Footnotes

1 http://mev.tm4.org/

2 http://software.broadinstitute.org/gsea/index.jsp

3 https://reactome.org/

4 www.cytoscape.org

## Abbreviations

simBOs: simplified brain organoids.
