# Supplementary material for: Simplified Brain Organoids for Rapid and Robust Modeling of Brain Disease
Source: Front Cell Dev Biol. 2020 Oct 28;8:594090. doi: 10.3389/fcell.2020.594090 (PMC7655657; doi:10.3389/fcell.2020.594090)
Supplement: Supplementary file 1 [file Data_Sheet_1.docx]

**Supplementary Materials**

**
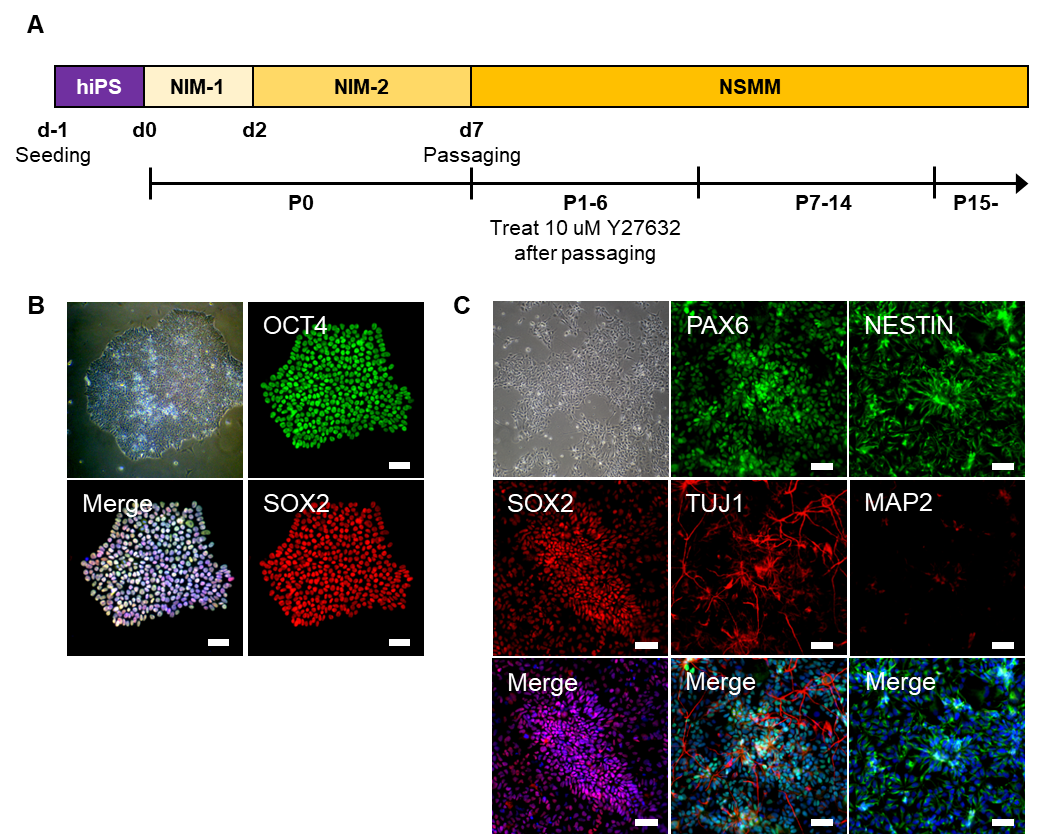
**

**FIGURE S1 |** Generation of primitive neural stem cells (pNSCs) from human iPSCs. **(A)** pNSC induction schematics. **(B and C)** Immunofluorescence analysis with pluripotency markers (OCT-3/4 and SOX2) markers, neural progenitor markers (SOX2, PAX6, and NESTIN), an immature neuronal marker (TUJ1) and mature neuronal marker (MAP2). Scale bars represent 100 µm.


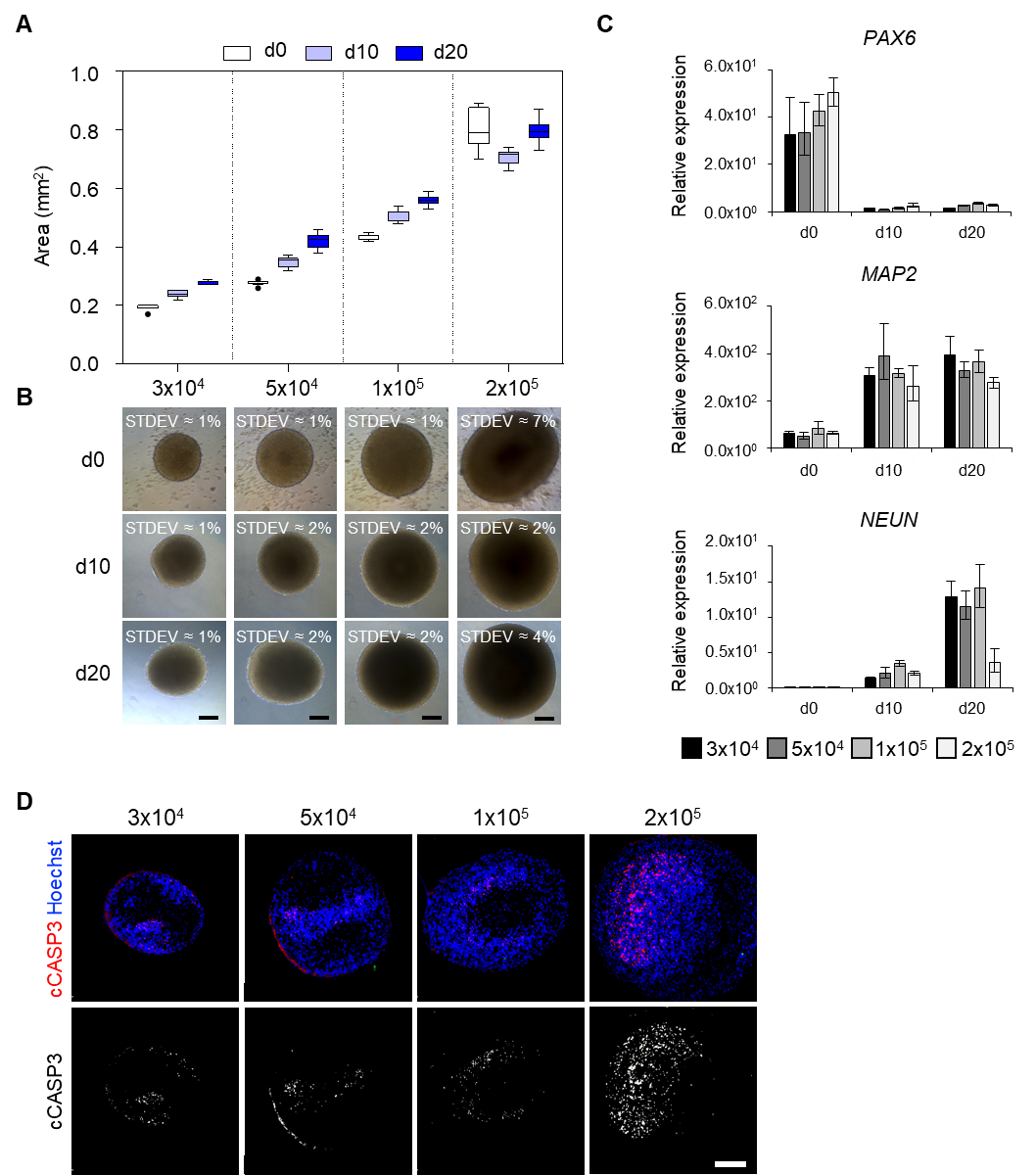


**FIGURE S2 |** Optimization of the number of starting cells for simBOs generation. **(A)** Quantification of the area (measured on phase-contrast images shown in S2B using Image J) in simBOs at day 0, 10, and 20 with the indicated number of starting cells. Data represent mean ± SE (n=10). **(B)** Representative images of area measurements in S2A. The standard deviation (STDEV) of the area was presented. Scale bars represent 200 µm. **(C)** qRT-PCR analysis for neural progenitor marker (*PAX6*) and neuronal markers (*MAP2* and *NEUN*) in simBOs at day 0, 10, and 20. Gene expression has been normalized to iPSCs. Data represent mean ± SE. **(D)** Immunofluorescence analysis of simBOs with apoptotic cell marker (cleaved caspase III; cCASP3). Scale bars represent 100 µm.

**FIGURE S3** **|** Robust production of simplified brain organoids (simBOs). **(A)** Graphical schematics for robust production of simBOs. **(B)** Phase contrast images of simBOs at day 10 of spontaneous differentiation from three independent biological replicates. Scale bars represent 200 µm. **(C)** Quantitative representation of the area of simBOs. The number of simBOs used in the analyses and the respective standard deviation/average (STDEV) of the area is shown for each batch.

**
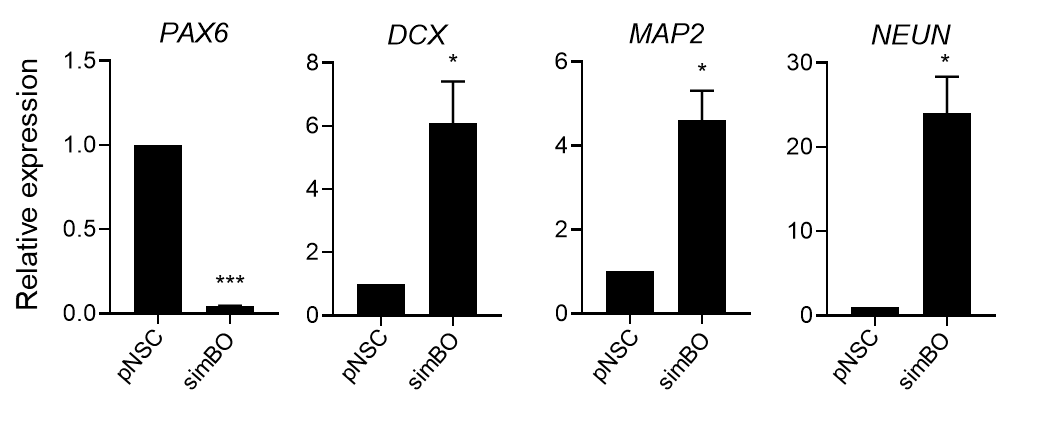
**

**FIGURE S4 |** Reproducibility of simBO production. Quantitative reverse transcription polymerase chain reaction (qRT-PCR) analysis for the indicated markers. Expression value was normalized to those of human pluripotent stem cells (hPSCs)-derived primitive neural stem cells (pNSC). Data represent mean ± standard error (SE) from the three batches, three technical replicates. Statistical significance of **p* < 0.05, ****p* < 0.001 was calculated by unpaired *t*-test.

**
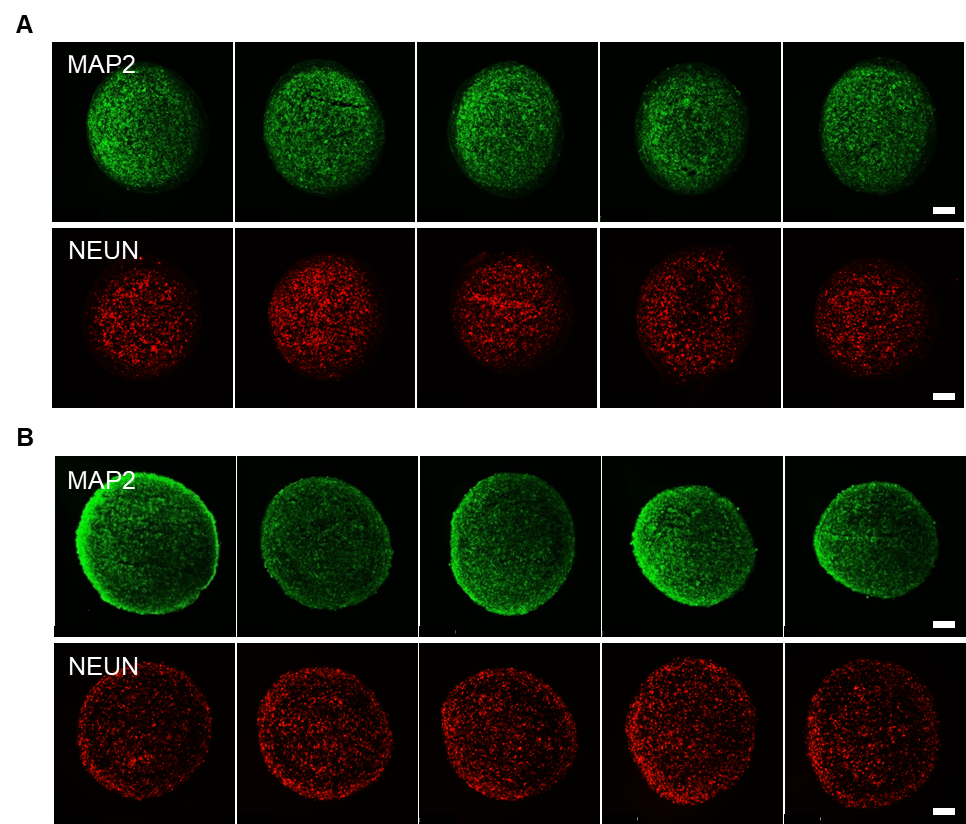
**

**FIGURE S5 |** Homogeneity of simBOs in neuronal marker expression. **(A-B)** Immunofluorescence analysis for mature neuronal markers (MAP2 and NEUN) in simBOs after spontaneous differentiation day 10 (A) or 20 (B). Scale bars represent 100 µm.

**FIGURE S6 |** Comparison of simplified brain organoids (simBOs) production from various cell lines. **(A–B)** Phase contrast and quantitative representation of the area of simBOs. The number of simBOs used in the analyses and the respective standard deviation/average (STDEV) of the area are shown, as well as immunofluorescence images for MAP2, GAFAP, and TUJ1. All analyses were conducted similarly in two cell lines: Parkinson’s disease (PD)-control and PD-patient at day 10 of spontaneous differentiation. Scale bars represent 200 µm. **(C)** Quantitative reverse transcription polymerase chain reaction (qRT-PCR) analysis of the indicated markers was performed in three cell lines of healthy control (AG), PD-control (Corr), and PD-patient (GS) at day 10 of spontaneous differentiation. Gene expression was normalized to induced pluripotent stem cells (iPSCs). Data represent mean ± standard error (SE) from three technical replicates.

**FIGURE S7 |** Gene Ontology (GO) analysis according to cellular component for each clusters of differentially expressed genes between 2D and 3D differentiation. Bar chart was presented of -Log10(P-value) of GO analysis.

**FIGURE S8 |** Heat map of representative reactome pathways enriched in 3D differentiation.

**FIGURE S9 |** Heat map of representative reactome pathways enriched in 2D differentiation.

**FIGURE S10 |** Comparison of 74 differentially expressed genes related to ECM organization during *in vitro* neural differentiation and *in vivo* brain development. **(A)** Flow chart depicting the comparative analysis process of gene expression of ECM organization. **(B)** Scatter gene expression plot comparing fold changes of the 74 differentially expressed genes during the 2D cultures and brain development or simBOs and brain development. **(C)** Heat map of the 74 differentially expressed genes during *in vitro* neural differentiation and *in vivo* brain development.

**
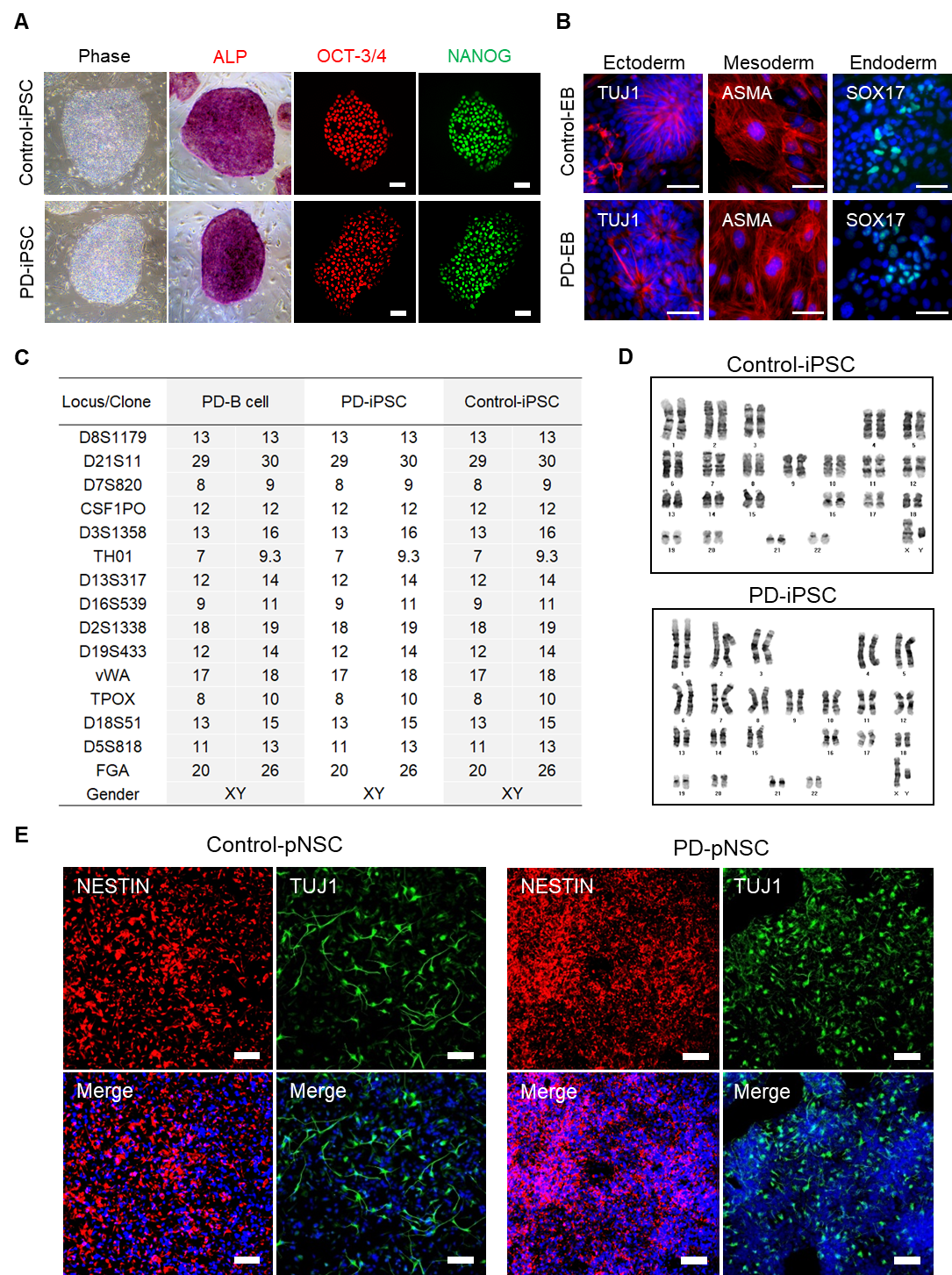
**

**FIGURE S11** | Characterization of Parkinson’s disease (PD) patient-derived and control iPSCs/ pNSCs. **(A)** Phase contrast, alkaline phosphatase expression, and representative immunofluorescence analysis with pluripotency markers OCT-3/4 and NANOG in iPSCs. Scale bars represent 100 µm. **(B)** In vitro differentiation via embryonic body (EB) formation of control-iPSCs and PD-iPSCs. Immunofluorescence analysis for ectodermal marker (TUJ1), mesodermal marker (ASMA) and ectodermal marker (SOX17). Scale bars represent 100 µm. **(C)** Karyotype analysis of control-iPSCs and PD-iPSCs. **(D)** STR analysis of Control-iPSCs, PD-iPSCs, and donor B cells for PD-iPSCs. (E) Immunofluorescence analysis with neural progenitor marker (NESTIN) and immature neuronal marker (TUJ1) in pNSCs. Scale bars represent 100 µm.

**Table S1 | List of primers used in this study**

| Quantitative PCR | |  |
| --- | --- | --- |
| **Gene** | **Forward primer** | **Reverse primer** |
| *PAX6* | GTCCATCTTTGCTTGGGAAA | TAGCCAGGTTGCGAAGAACT |
| *TUBB3* | GGCCAAGGGTCACTACACG | GCAGTCGCAGTTTTCACACTC |
| *DCX* | TCCCGGATGAATGGGTTGC | GCGTACACAATCCCCTTGAAGTA |
| *MAP2* | CGAAGCGCCAATGGATTCC | TGAACTATCCTTGCAGACACCT |
| *NEUN* | CCCATCCCGACTTACGGAG | GCTGAGCGTATCTGTAGGCT |
| *SYN1* | AGTTCTTCGGAATGGGGTGAA | CAAACTGCGGTAGTCTCCGTT |
| *GRIN1* | ACCCCAAGATCGTCAACATTG | GGCTAACTAGGATGGCGTAGA |
| *GRIN2B* | GTCCCTGGACGATGGAGATTC | CAGTCAGCCCTACTGAGTTGG |
| *S100B* | TGGCCCTCATCGACGTTTTC | ATGTTCAAAGAACTCGTGGCA |
| *TH* | GGGCGTTGTAAGCAGAACG | AAGGCCCGAATCTCAGGCT |
| *EN1* | CGCAGCAGCCTCTCGTATG | CCTGGAACTCCGCCTTGAG |
| *YAP1* | TAGCCCTGCGTAGCCAGTTA | TCATGCTTAGTCCACTGTCTGT |
| *SOX2* | GCCGAGTGGAAACTTTTGTCG | GGCAGCGTGTACTTATCCTTCT |
| *TEAD1* | ATGCCAACCATTCTTACAGTGAC | ACAGTTCCTTTAAGCCACCTTTC |
| *TEAD2* | CTTCGTGGAACCGCCAGAT | GGAGGCCACCCTTTTTCTCA |
| *CDC20* | GACCACTCCTAGCAAACCTGG | GGGCGTCTGGCTGTTTTCA |
| *MCM5* | AGCATTCGTAGCCTGAAGTCG | CGGCACTGGATAGAGATGCG |
| *RPL7* | CCAAATTGGCGTTTGTCAG | GCATGTTCGAAGCCTTGTTG |

**TABLE S2 |** **List of antibodies used in this study**

| Immunofluorescence (*secondary antibodies) | |  |  |
| --- | --- | --- | --- |
| **Antibody** | **Supplier** | **Product number** | **Dilution** |
| Rabbit anti-OCT3/4 | Santa Cruz Biotechnology | sc-5279 | 1:300 |
| Goat anti-SOX2 | R&D systems | MAB2018 | 1:200 |
| Rabbit anti-PAX66 | BioLegend | PRB-278p-100 | 1:250 |
| Mouse anti-NESTIN | R&D systems | MAB1259 | 1:500 |
| Mouse anti-Ki-67 | BD Biosciences | 556003 | 1:200 |
| Rabbit anti-Cleaved Caspase-3 | Cell Signaling Technology | 9661-S | 1:300 |
| Mouse anti-TUJ1 | BioLegend | 802001 | 1:2000 |
| Chicken anti-MAP2 | Abcam | ab5392 | 1:50000 |
| Mouse anti-NEUN | R&D systems | MAB377 | 1:50 |
| Goat anti-GFAP | Dako | Z0334 | 1:500 |
| Rabbit anti-SYN1 | Millipore | AB1543 | 1:1000 |
| Rabbit anti-YAP/TAZ | Cell Signaling Technology | 8418S | 1:100 |
| Mouse anti-TH | Sigma-Aldrich | T1299 | 1:10000 |
| *AlexaFluor 594-conjugated chicken anti-rabbit IgG | Invitrogen | A21442 | 1:500 |
| *AlexaFluor 594-conjugated donkey anti-mouse IgG | Invitrogen | A21203 | 1:500 |
| *AlexaFluor 488-conjugated donkey anti-rabbit IgG | Invitrogen | A21206 | 1:500 |
| *AlexaFluor 488-conjugated donkey anti-mouse IgG | Invitrogen | A21202 | 1:500 |
| *AlexaFluor 488-conjugated chicken anti-goat IgG | Invitrogen | A21467 | 1:500 |
| *Cy3-conjugated donkey anti-chicken IgY | Millipore | AP194C | 1:500 |
| Hoechst33342 | Invitrogen | H-21492 | 1:2000 |
| Immunoblot (*secondary antibodies) | |  |  |
| **Antibody** | **Supplier** | **Product number** | **Dilution** |
| Rabbit anti-phospho-LRRK2 (S1292) | Abcam | ab203181 | 1:1000 |
| Rabbit anti-total LRRK2 | Abcam | ab133449 | 1:1000 |
| Rabbit anti-phospho-RAB10 (T73) | Abcam | ab230261 | 1:1000 |
| Rabbit anti-total RAB10 | Cell Signaling Technology | 8127T | 1:1000 |
| Mouse anti-TH | Sigma-Aldrich | T1299 | 1:10000 |
| Chicken anti-MAP2 | Abcam | ab5392 | 1:50000 |
| Rabbit anti-LC3B-I/II | Cell Signaling Technology | 2775S | 1:1000 |
| Mouse anti-β-actin | Sigma-Aldrich | A5441 | 1:20000 |
| *HRP-linked anti-mouse IgG | Cell Signaling Technology | 7076S | 1:5000 |
| *HRP-linked anti-rabbit IgG | Cell Signaling Technology | 7074S | 1:5000 |
| *HRP-linked anti-chicken IgY | Thermo Fisher Scientific | SA1-300 | 1:5000 |
